# Space/Population and Time/Age in DNA methylation variability in humans: a study on IGF2/H19 locus in different Italian populations and in mono- and di-zygotic twins of different age

**DOI:** 10.18632/aging.100476

**Published:** 2012-07-31

**Authors:** Chiara Pirazzini, Cristina Giuliani, Maria Giulia Bacalini, Alessio Boattini, Miriam Capri, Elisa Fontanesi, Elena Marasco, Vilma Mantovani, Michela Pierini, Elisa Pini, Donata Luiselli, Claudio Franceschi, Paolo Garagnani

**Affiliations:** ^1^ CIG - Interdipartimental Center L. Galvani, University of Bologna, Bologna, 40126, Italy; ^2^ Department of Experimental Pathology, University of Bologna, Bologna, 40126, Italy; ^3^ Department of Experimental Evolutionary Biology, University of Bologna, Bologna, 40126, Italy; ^4^ CRBA - Applied Biomedical Research Center, S. Orsola-Malpighi Polyclinic, Bologna, 40138 Italy

**Keywords:** DNA methylation, IGF2/H19, twins, aging, population epigenetics

## Abstract

Little is known about the impact of space (geography/ancestry) and time (age of the individuals) on DNA methylation variability in humans. We investigated DNA methylation of the imprinted *IGF2/H19* locus in: i) a cohort of individuals homogeneous for age and gender (males with restricted age range: 30-50 years) belonging to four Italian districts representative of the major genetic clines, informative for the geographical dimension; ii) a cohort of monozygotic (MZ) and dizygotic (DZ) twins of different ages (age-range: 22-97 years), informative for the temporal dimension. DNA methylation of the analyzed regions displayed high levels of inter-individual variability that could not be ascribed to any geographical cline. In MZ twins we identified two *IGF2/H19* regions where the intra-couple variations significantly increased after the age of 60 years. The analysis of twins’ individual life histories suggests that the within twin pairs difference is likely the result of the aging process itself, as sharing a common environment for long periods had no effect on DNA methylation divergence. On the whole, the data here reported suggest that: i) aging more than population genetics is responsible for the inter-individual variability in DNA methylation patterns in humans; ii) DNA methylation variability appears to be highly region-specific.

## INTRODUCTION

DNA methylation is widespread across the genomes of different organisms and in mammals usually consists in the enzymatic addition of a methyl group to the carbon-5 of cytosine ring of a CpG dinucleotide. Through the recruitment of methyl-binding proteins, this modification induces an inactive chromatin structure that represses transcription. While the bulk of human genome is generally methylated, the promoters of around 40% of genes contain CpG-rich regions, termed CpG islands, whose methylation status is strictly regulated during development and cellular differentiation [1-3]. Deregulation in methylation patterns can lead to disease onset. More recently it has been shown that tissue- and disease-specific differentially methylated regions (DMR) are more frequent in CpG island shores rather than in CpG islands [4,5].

Although DNA methylation is generally considered a stable modification, it is well established that methylation profiles vary in pathological conditions, and it is also accepted that external factors, such as diet, age, toxins and lifestyle, can induce quantitative hypo- or hyper- DNA methylation [6-13]. Only few studies have focused the effect of such factors on inter-individual variability of DNA methylation patterns among human populations and subjects [14-17]. Regarding time/age, variations in methylation levels of the *IGF2/H19* locus were observed in a cohort of newborn twins, both between individuals and within twin pairs [17]. In this study, dizygotic twins (DZ) resulted more discordant than monozygotic twins (MZ), suggesting that a heritable component can affects the epigenetic status of this locus. Moreover, Heijmans and colleagues [16] analyzed the variations in DNA methylation of the same *IGF2/H19* locus in a cohort of adolescent and middle-aged twins (13-62 years of age) and demonstrated that a substantial part of the variation observed across individuals was ascribable to heritable factors and single nucleotide polymorphisms (SNPs) *in cis*, rather than to the cumulative effect of environmental and stochastic factors occurring with age. Although this study involved a high number of twins (N=372), it did not include old twin pairs, and therefore could not appreciate the possible epigenetic variability of the locus occurring later in life (after sixties).

Regarding space/population, only few studies addressed the DNA methylation variability at the population level (*population epigenetics*) [18], despite its great potential interest owing to the interaction of both environmental and genetic variables in determining the DNA methylation architecture. To our knowledge, the only two available studies have investigated DNA methylation in multiethnic cohorts of healthy women [19] and of men affected by prostate cancer [20].

To better understand the intricate relationship between spatial (geography/ancestry) and temporal (age of the individuals) dimensions on DNA methylation variability, we took advantage of two *ad hoc* models: i) Cohort 1, constituted of 376 individuals homogeneous for gender (males) and age (age range 30-51), but differing for ancestral geographical origin and place of living, i.e four Italian regions (Northern, Central and Southern Italy, and Sardinia); ii) Cohort 2, constituted of 31 monozygotic (MZ) and 16 dizygotic (DZ) twin couples of different ages, spanning from 22 to 97 years, homogeneous for geographic origin (Northern Italy, Bologna area). Both the models were analyzed for variations in DNA methylation status of 4 target regions in the imprinted *IGF2/H19* locus, previously analyzed by other groups [16,17].

## RESULTS

### Characterization of the *IGF2/H19* target regions

**Figure 1:**
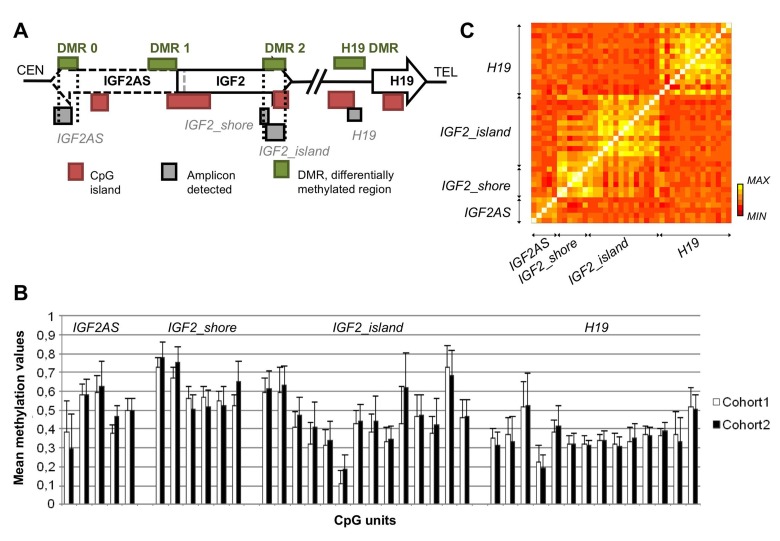
CpGs spatial localization and their methylation patterns (**A**) Spatial disposition of the amplicon analyzed in relation to functional elements. (**B**) Mean methylation level with the corresponding standard deviation for each CpGs considering the whole dataset. (**C**) Correlation matrix of CpG sites of *IGF2_shore, IGF2AS, H19* and *IGF2_island* amplicons.

Four target regions (amplicons) in the *IGF2/H19* locus were analyzed: *IGF2AS*, 3 kb from a CpG island, in exon 3 of *IGF2AS* transcript, within the *IGF2* DMR 0; *H19*, upstream the transcription start site of *H19* gene, partially overlapping a CpG island (*H19* DMR); *IGF2_island*, within a CpG island in the last exon of *IGF2* gene (DMR 2); *IGF2*_shore, in the shore upstream the island targeted by the *IGF2_island* amplicon (Figure 1A). *IGF2AS* and *H19* have been previously analyzed by Heijmans et al. [16] and Ollikainen et al. [17], while *IGF2_island* and *IGF2_shore* were analyzed here for the first time.

### Mean methylation levels and inter-individual variability of *IGF2/H19* locus

Figure 1B reports mean methylation values and their standard deviations for the 4 target regions considering cohort 1 and 2 separately. For cohort 1 the mean DNA methylation levels observed in *IGF2AS, IGF2_shore, IGF2_island* and *H19* were 49%, 60%, 43% and 36% respectively. These values are similar for cohort 2, where the mean methylation values in *IGF2AS, IGF2_shore, IGF2_island* and *H19* were 49%, 62%, 47% and 36%. Notably *IGF2AS* and *H19* DNA methylation values were comparable to those previously reported in literature [16,17].

Considerable inter-individual variation in DNA methylation values was observed within each CpG unit. Within a single amplicon, DNA methylation values of the CpG units were strongly correlated with each others, while correlation values were lower between CpGs belonging to different amplicons (Figure 1C). Intermediate correlation levels were observed only between *IGF2_island* and *IGF2_shore*, as expected because of their adjacent chromosomal position.

### Space/ancestry: geographical dimension

**Figure 2:**
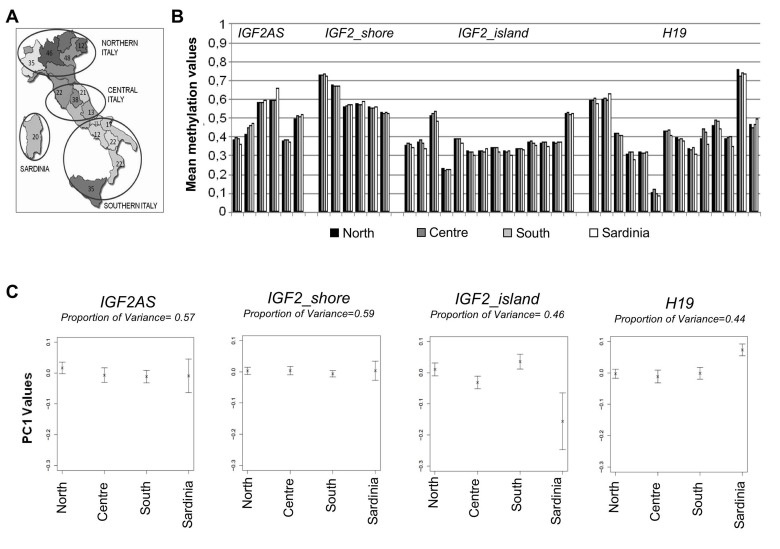
Methylation variation in cohort 1 (**A**) Number of samples from different Italian districts and corresponding groups made for the statistical analysis. (**B**) Mean methylation levels of each CpGs unit in the 4 macroarea. (**C**) PC1 values plotted for each macro-area and the corresponding percentage of variance.

The Italian cohort comprises 376 middle-aged males (age range: 30-51 years) representative of the four main Italian geographical areas: Northern Italy (141 samples), Central Italy (94 samples), Southern Italy (121 samples) and Sardinia (20 samples) (Figure 2A). We evaluated if the observed variability in methylation levels of the *IGF2/H19*locus could be explained by ancestry and place of living of the four populations studied.

Mean methylation levels of each CpG unit were calculated for the 4 geographical areas (Figure 2B). The results showed that the methylation patterns were comparable throughout all the samples. No evident differences were detectable according to sample ancestry/geography, except for few CpGs (*i.e.* CpG 20 of *IGF2_island* amplicon).

Principal component analysis (PCA) was used to capture the most salient patterns of variation in each amplicon for the 4 geographical areas. The percentages of variance explained by principal component 1 (PC1) values are reported in Figure 2C. No statistically significant difference was observed between PC1 values, as confirmed by analysis of variance (ANOVA) (Table 1).

**Table 1:** *p_values* from ANOVA on PC1 values calculated based on DNA methylation levels in samples belonging to different Italian districts

	ANOVA – *p_values*
***IGF2_shore***	0.93
***IGF2_island***	0.45
***IGF2AS***	0.80
***H19***	0.44

### Time/age: temporal dimension

**Figure 3:**
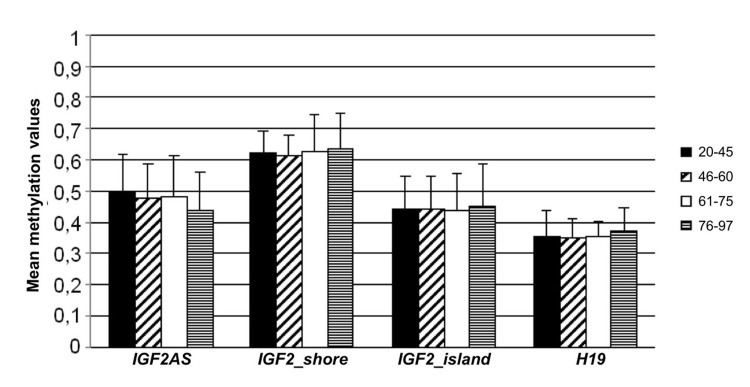
Methylation variation in cohort 2 The twins cohort was divided in 4 age classes (20-45, 46-60, 61-75, 76-97). Mean methylation values and the corresponding standard deviation for the 4 analyzed amplicons (*IGF2AS, IGF2_shore, IGF2_island* and *H19*) are reported for each age-class.

The twins cohort (cohort 2) includes 94 twins (62 MZ and 32 DZ) with age ranging from 22 to 97 years all recruited in the area of Bologna (Emilia Romagna, Italy). Cohort 2 was divided into four age classes (20-45, 46-60, 61-75, 76-97) and for each amplicon, mean methylation value and mean standard deviation were calculated (Figure 3). No significant variations in mean methylation levels were observed. In the first 2 age classes IGF2_shore was characterized by a minor range of variation if compared to the other amplicons. Moreover, while mean standard deviations in *IGF2_island*, IGF2AS and H19 were stable over the time, *IGF2_shore* showed a doubling in their values in the older age classes.

**Figure 4:**
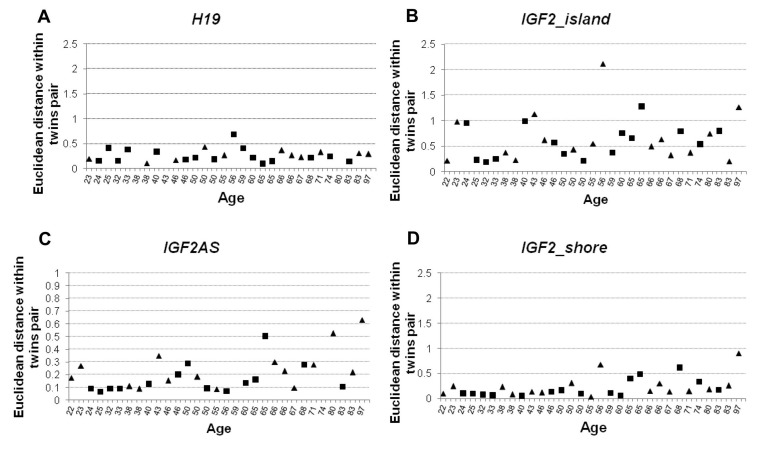
Pearson correlation values calculated within twin pairs in the 4 analyzed amplicons (**A**) Correlation values within MZ twin couple in *H19* amplicon. (**B**) Correlation values within MZ twin couple in *IGF2_island*. (**C**) Correlation values within MZ twin couple in IGF2AS. (**D**) Correlation values within MZ twin couple in *IGF2_shore* amplicon. In each figure male individuals are indicated with squares and female using a triangle.

In order to estimate DNA methylation variability within the MZ twins couples, Pearson's correlation and Euclidean distance values for each twins pair were calculated. Pearson's correlation was calculated considering methylation values of each CpG from the same amplicon. The correlation values were then plotted against the age of the subjects (Figure 4). H19 was characterized by a high degree of within-twin pairs correlation (Figure 4A) which was maintained up to old ages (Spearman's correlation with age ρ= −0.094). *IGF2_island* also did not display variations of correlation levels with age (Spearman's correlation with age ρ = −0.105). However, in this case the intra-couple correlation scores were lower, indicating that high levels of variability characterize the CpG island of *IGF2* (Figure 4B). Interestingly, both *IGF2AS* and *IGF2_shore* showed an age dependent trend (Spearman's correlation ρ = −0.613 and r = −0.651 respectively), with high correlation levels till the age of 50, followed by a progressive decrease of correlation in the couples of older age (Figure 4C-4D). A possible threshold between the age of 56 and 60 years can be envisaged, and after such age limit the values of intra-couple correlation levels are much more scattered.

Then, the Euclidean distance between the CpG units of each amplicon. was calculated for each MZ twins pair.

**Figure 5:**
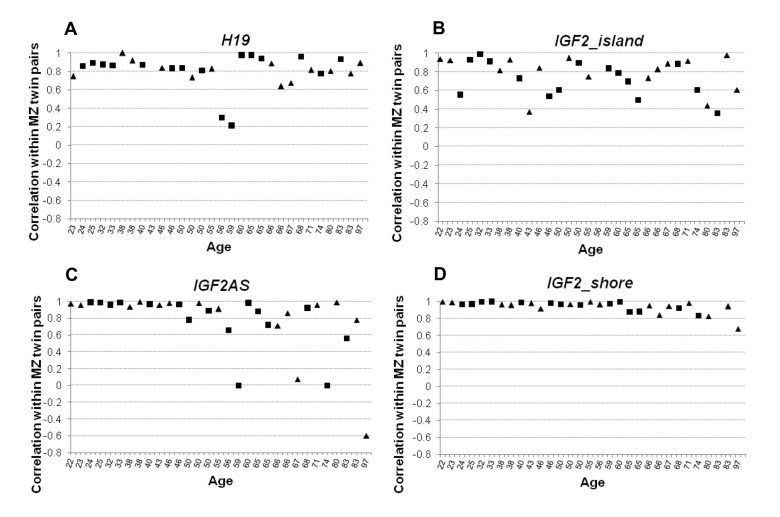
Euclidean distance calculated using DNA methylation values within MZ twin pairs considering the 4 considered amplicons (**A**) Distance within twin pairs in *H19* amplicon. (**B**) Distance values within twin pairs in *IGF2_island* amplicon. (**C**) Distance values within twin pairs in *IGF2AS* amplicon. (**D**) Distance values within twin pairs in *IGF2_shore* amplicon. In each figure male individuals are indicated with squares and female using a triangle.

Regarding *H19* and *IGF2_island* the distance values did not show any age-dependent trend (Figure 5A and 5B, Spearman's correlation r = 0.099 r = 0.216 respective-ly). On the contrary, *IGF2AS* and *IGF2_shore* (Figure 5C and 5D) showed an age-dependent increase in the intra-couple Euclidean distances (Spearman's correlation ρ = 0.462 and r= 0.553 respectively), with a scatter of the distance values after the age threshold of 60, as reported for Pearson's correlation analysis.

The data were also subdivided according to sex to exclude an influence on the patterns of correlation and distance values (Figure 4 and Figure 5).

To confirm the previous observations, samples from cohort 2 were divided into 2 groups according to age (below and over 60 years old) and Student t-test on correlation and Euclidean distance values was performed. Significant differences between the 2 groups were found only for *IGF2_shore* and *IGF2AS* amplicons (Table 2).

**Table 2:** Student t-test on correlation and Euclidean distance scores using 60 years old as age threshold

Amplicon	Age limit	Values considered	*p_values*
*IGF2_shore*	60	Correlation	0.00047***
*IGF2AS*	60	Correlation	0.022*
*H19*	60	Correlation	0.27
*IGF2_island*	60	Correlation	0.77
*IGF2_shore*	60	Euclidean distance	0.0075**
*IGF2AS*	60	Euclidean distance	0.023*
*H19*	60	Euclidean distance	0.48
*IGF2_island*	60	Euclidean distance	0.57

### Evaluation of heritability and environmental influences on DNA methylation

MZ and DZ twins were used to estimate the genetic and environmental influences on DNA methylation of the *IGF2/H19* locus.

**Figure 6:**
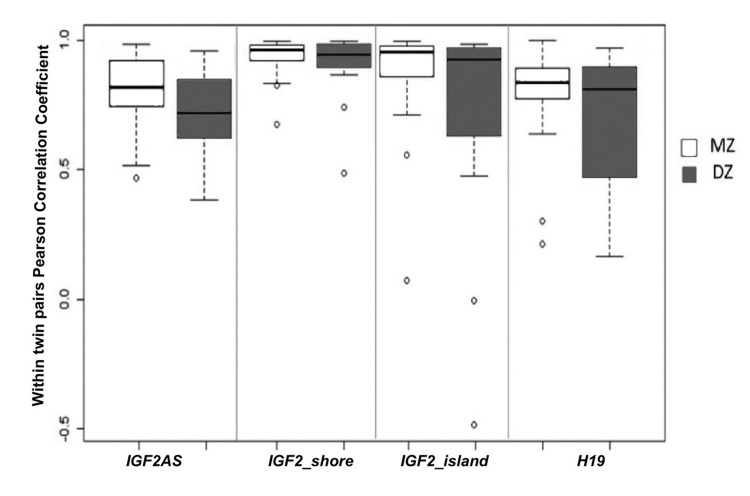
Comparison between intra-couple correlation (ICC) in MZ and DZ twins Boxplot of ICC for each amplicon dived on the basis of zygosity.

**Figure 7:**
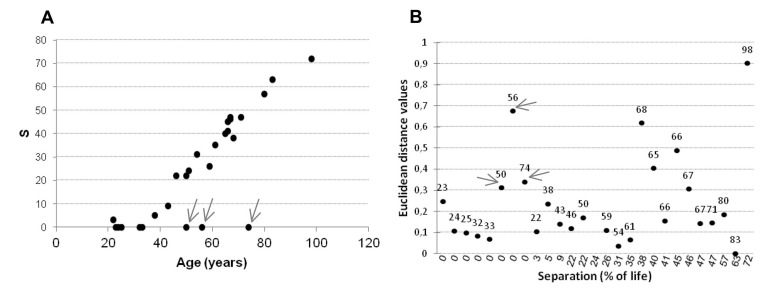
Analysis of twins individual histories (**A**) Percentage of years lived separately for each twin pairs in relation to their ages. (**B**) Euclidean distance within twin pairs related to years the twins lived separately in *IGF2_shore* amplicon. S = (years lived in a different house / age)*100

Firstly for each amplicon intra-couple levels of correlation between MZ and DZ twins were compared. Like MZ, also DZ twins showed high values of correlation in the 4 amplicons (Figure 6), suggesting a strong epigenetic control on this locus. In *IGF2AS, H19* and *IGF2_island*, the intra-couple correlation in MZ was higher compared with that of DZ. Only in*IGF2_shore* median correlation values were similar among MZ (r=0.94) and DZ (r=0.90) twins. Falconer method confirmed this observation, showing that IGF2_shore is more influenced by environmental factors (h2=0.07) when compared with *IGF2AS* (h2=0.37), *H19* (h2=0.34) and *IGF2_island* (h2=0.20). As *IGF2_shore* methylation seems to be more affected by environmental factors, the total lifetime in which the twins did not shared the same environment (i.e. the total lifetime in which they did not live in the same house) was considered. As expected, an increase in percentage of life in which the twins lived separated (S = (years lived in a different house / age)*100) was observed for older couples (Figure 7A).

Low Euclidean distance levels characterized twins with small S values, while a scatter after the threshold of*S*=35, which in our cohort corresponds to the age of 60, was observed (Figure 7B). There were 3 main exceptions, indicated with an arrow in Figure 7B. These subjects (aged 50, 56 and 74) were still living in the same house at the time of recruitment (small S values) but were characterized by high Euclidean distance values for *IGF2_shore*.

## DISCUSSION

Our results indicate that the 4 analyzed regions in the *IGF2/H19* locus show high inter-individual variability both in cohort 1 (spatial/population dimension) and in cohort 2 (temporal/aging dimension). *IGF2AS* and *H19* have mean methylation values comparable to previous reports [16,17], indicating that methylation profiles are consistent among different populations and that the experimental technique used for methylation analysis is highly reproducible between independent laboratories. On the contrary, *IGF2_island* and *IGF2_shore* were analyzed for their methylation values here for the first time.

Previous studies have analyzed methylation variability in multiethnic populations, but the enrolled subjects lived in the same geographical area and were therefore exposed to the same environmental factors [19, 20]. On the contrary, cohort 1 was selected in order to include individuals both belonging to different genetic clines and living in their original geographical area. Our results show that methylation variability in cohort 1 cannot be explained on the basis of the geographic provenience of subjects. This implies that the genetic structure of the Italian population does not influence the methylation pattern of the analyzed loci. At the same time, it means that there are no environmental cues specifically associated with Italian macro-areas able to generate clear population epigenetic signatures in the *IGF2/H19* locus.

Methylation of IGF2 promoter has been shown to increase with aging [21]. On the contrary, mean methylation values of the 4 regions that we considered in *IGF2/H19* locus do not change according to age in cohort 2. This suggests that methylation levels of different regions in the same locus are differently affected by the aging process. At the same time, in young individuals from cohort 2, DNA methylation variations is smaller in *IGF2_shore* respect to the other analyzed amplicons indicating a stricter control of its methylation levels. Accordingly previous studies have highlighted that the range of methylation variation depends on the genomic position [14]. Moreover, only in *IGF2_shore* the range of methylation values increases in the elderly suggesting a clear susceptibility of this region to epimutations that occur with aging.

MZ twins model allowed the investigation of the maintenance of DNA methylation profiles during the lifespan. *IGF2AS* and *IGF2_shore* methylation levels were highly concordant within twin pairs, but only until the 60 years age threshold. Homogeneous methylation values in *IGF2AS* and *IGF2_shore* until the age of 60 years can be in part due to the genetic identity and the sharing of intrauterine and neonatal environments. It is interesting to observe that the threshold of 60 years coincides with the end or with the significant reduction of the reproductive capacity of individuals. A loss in molecular fidelity after the threshold of 60 years has been described [22]. This can affect also the DNA methylation machinery, impairing its ability to maintain the methylation patterns across cellular divisions [23]. The extent and the rate of this process can be influenced by the genetic background of subjects [24], accordingly in our cohort, a subset of couples maintains the concordance in DNA methylation profiles also at old age.

Comparison between MZ and DZ twins confirms a genetic component in the variability observed in *IGF2/H19* locus in our cohort. These analysis highlight also an effect of non-genetic factor, which is stronger for *IGF2_shore*. This observation is supported by the 3 old twins pairs that had always lived together, in the same house, sharing the same daily habits. They show high distance values in *IGF2_shore*, similar to those observed in the old couples that lived separately. This data suggests that age-related epimutations are independent from the shared environment and are more likely related to individual histories or stochasticity. A larger sample of old twins that lived together should be considered to confirm this hypothesis.

Previous data show that different imprinted genes have different ability to maintain their methylation status [25]. Our results not only confirm these observations, but also show that within the same imprinted locus there are regions whose methylation maintenance is differently influenced by aging.

It is interesting to highlight that, between the 4 regions that we have analyzed in *IGF2/H19* locus, *IGF2_shore* is the one in which the scatter in methylation variability during aging is more evident. This result confirms recent evidences that indicate CpG shores as the regions most variable in terms of methylation levels across normal and pathological tissues and between different cell types [4].

The ability of imprinted genes to maintain their methylation profile has been proposed as a marker of epigenetic stability [25]. In fact, although imprinted loci are under a strict epigenetic control, a relaxation in their regulation can be observed both in pathological [26] and normal conditions [27]. Further studies are needed to deepen our knowledge on age-related DNA methylation variability of imprinted genes, with specific attention to the shore elements.

In conclusion our data confirm the loss of control over DNA methylation maintenance with age only in specific genomic regions (shore). In particular we observe a sudden increase in intra-couple variability from the age of 60, when the reproductive capacities decline and, as a consequence, the effect of natural selection falls off. On one side this decline could be interpreted as a loss of capacities of transmitting the methylation patterns across cellular divisions. On the other side, as the weaker epigenetic control can result in a reduction of gene expression control, it is possible to speculate that this mechanism could represent a molecular strategy to counteract the physiological fitness decline that occurs during aging by offering a wider range of expression possibilities.

## MATERIALS AND METHODS

### Ethics statement

Informed consent was obtained from all partecipants and was approved by the Medical Ethics Committee of the S. Orsola-Malpighi Polyclinic (Bologna, Italy).

### Samples

*Twins:* The twins cohort was recruited between 2004 and 2005 in Bologna and neighboring districts to reduce possible bias associated to different geographical origin of the samples. The cohort comprises 47 twin pairs (31 MZ an 16 DZ pairs) with a mean age of 55 years (age range: 22-97 years; Table 3). *Italian population:* in order to assemble the most representative and informative Italian sample, with an ancient regional ancestry in the area, and an adequate coverage for both the number of populations and the number of individuals within each population sample, an appropriate and accurate sampling strategy was built based on a preliminary surname-based study (Boattini et al., submitted).

**Table 3:** Cohort 1 characteristics

	Twins		All
	Monozygotic	Dizygotic	Total samples
**N**	62 (31 pairs)	32 (16 pairs)	94 (47 pairs)
**Mean age** **(SD; range)**	54.0 (19.4; 22-97)	57.8 (12.8;36-79)	55.3 (17.4; 22-97)
**Male (%)**	32 (51.6%)	20 (62.5%)	52 (55.3%)

Groups of homogeneous provinces (“sampling areas”) were aggregated according to the surname-based clusters. Provinces (“sampling points”) were selected within each sampling area according to the complex geographic and historical Italian backgrounds. Individuals (“samples”) were chosen, within each province, based on two different strategies, the “grandparents criterion” and “founder surnames analysis“, so that were included into the study only those individuals whose four grandparents were born in the same sampling area, taking also into account the presence of founder surnames.

Using this sampling strategy 378 male individuals from 29 provinces scattered in 15 regions of Italy were collected (Figure 2A).

### DNA methylation measurement and associated data cleaning

The level of DNA methylation was measured on genomic DNA using a MALDI-TOF mass spectrometry-based method (Epityper, Sequenom, San Diego, CA) as previously described [28]. DNA was extracted from whole blood (QIAamp 96 DNA Blood Kit, Qiagen, Hilden, Germany), quantified using Picogreen (Quant-iT dsDNA Broad-Range Assay Kit Invitrogen, Carlsbad, CA), and 1 μg was treated with sodium bisulfite using the EZ methylation kit (Zymo-Research, Irvine, CA). The treatment converts non-methylated cytosine into uracil, leaving methylated cytosine unchanged. In this way variations in the sequence are produced depending on DNA methylation status of the original DNA molecule. PCR amplification, addition of SAP solution and Transcription/RNase A cocktails were performed according to the protocol provided by Sequenom and the mass spectra were analyzed by EpiTYPER analyzer (Sequenom, San Diego, CA). *IGF2_shore* amplicon encompassed 276 bp (NCBI build 36, chr11: 2 111 039 - 2 111 314). IGF2AS encompassed 338 bp (NCBI build 36, chr11: 2 126 035 - 2 126 372). H19 encompassed 413 bp (NCBI build 36, chr11: 1 975 948 - 1 976 360). IGF2_island encompassed 453 bp (NCBI build 36, chr11: 2 111 119 - 2 110 666). The primers sequences used to amplify this regions are listed in Table 4.

**Table 4:** CpG Units considered after data cleaning and primers used to amplify the 4 target regions

Amplicon Name	CpG sites after data cleaning	Total Cpg Units	Primers sequences
***IGF2_shore***	CpG 1,2 – CpG 3,4 - CpG 6 - CpG 7 - CpG 8 - CpG 9,10,11	6	Forward: aggaagagagGAAGGGGTTGGTTAGTAGGTGTTTGT Reverse: cagtaatacgactcactatagggagaaggctCCTAAACCCCTTTCCCACTCTCTAA
***IGF2_island***	CpG 1,2 - CpG 4 - CpG 11,12 - CpG 13 - CpG 14 - CpG 15 - CpG 16 - CpG 17 - CpG 18,19 - CpG 20 - CpG 21 - CpG 25 - CpG 26 - CpG 27	14	Forward: aggaagagagTATAGGGGTGGTTTGTTAGGTTAGG Reverse: cagtaatacgactcactatagggagaaggcTAAATCAAAAAAAACCCCAAAAAAAC
***IGF2AS***	CpG 1 - CpG 3 - CpG 4 - CpG 6,7 - CpG 8	5	Forward: aggaagagagTGGATAGGAGATTGAGGAGAAA Reverse: cagtaatacgactcactatagggagaaggctAAACCCCAACAAAAACCACT
***H19***	CpG 1 - CpG 2 - CpG 6 - CpG 7 - CpG 8 - CpG 9,10 - CpG 12 - CpG 13 - CpG 14,15 - CpG 17 - CpG 18,19 - CpG 20 - CpG 22 - CpG 24	14	Forward: aggaagagagGGGTTTGGGAGAGTTTGTGAGGT Reverse: cagtaatacgactcactatagggagaaggctATACCTACTACTCCCTACCTACCAAC

In this way, a total of 50 CpG units in the 4 gene regions were interrogated for their methylation level. A rigorous data cleaning process was performed to remove unreliable measurements before statistical analysis. Firstly, DNA samples for which methylation level could be established for <60% of the CpG sites were removed. Secondly, CpG sites with more than 30% missing data points within an amplicon were not analyzed. Finally CpG sites that show a bimodal pattern due to the presence of a SNP were removed. Six CpG sites on four fragments could not be measured independently because the fragments had the same molecular weight and were overlapping in the spectrum. After data cleaning, we were able to analyze a total of 39 CpG units in the 4 genomic regions (Table 4).

### Statistical analysis

All analysis were performed using R (http://cran.r-project.org)/). Prior to statistical analysis, methylation data were checked for complete bisulfite conversion using the MassArray package [29]. h^2^ values were calculated according to Falconer's formula [30].
